# Theory of mind in remitted bipolar disorder: Interpersonal accuracy in recognition of dynamic nonverbal signals

**DOI:** 10.1371/journal.pone.0222112

**Published:** 2019-09-11

**Authors:** Usue Espinós, Enrique G. Fernández-Abascal, Mercedes Ovejero

**Affiliations:** 1 Facultad de Psicología, Universidad Nacional de Educación a Distancia, Madrid, Spain; 2 Facultad de Psicología, Universidad Complutense de Madrid, Madrid, Spain; National Institutes of Health, UNITED STATES

## Abstract

A relatively unexplored aspect in bipolar disorder (BD) is the ability to accurately judge other´s nonverbal behavior. To explore this aspect of social cognition in this population is particularly meaningful, as it may have an influence in their social and interpersonal functioning. The aim of this research was to study interpersonal accuracy (IPA) in remitted BDs, that is, the specific skills that fall under the general term *Theory of Mind* (ToM). Study participants included 119 remitted individuals with BD (70 BD I and 49 BD II), and they were compared with a group of 39 persons diagnosed with unipolar depression (UD) and 119 control participants. The MiniPONS was used to test the whole spectrum of nonverbal cues as facial expressions, body language and voice. Results indicated a superiority of the control group with statistically significant differences both in the performance in the MiniPONS (number of right answers) and in each of the areas evaluated by this test. BD groups, in recognition of the meaning of gestures in face, body and voice intonation, performed significantly worse than controls. ANCOVA analysis controlling the effect of age shows that control group performed significantly better compared to clinical groups, and there were no differences between UD and BD groups. The results indicate a deficit in IPA and suggest that better comprehension of deficiencies in interpersonal accuracy in BD may help to develop new training programs to improve in these patients the understanding of others, which might have a positive impact in their psychosocial functionality, and thus lead to the objective of functional rehabilitation.

## Introduction

Bipolar disorder (BD) is a psychiatric disorder characterized by periods of mania and depression [1]. Bipolar I (BD I) and II (BD II) are defined by a history of phases of elevated mood and a history of major depressive episodes, but BD II is distinguished from BD I by the presence of episode**s** of hypomania [1]. Despite treatment, many individuals with BD experience impaired functioning [2]. BD is associated with high rates of disability, with significant impairment in work, family and social life, beyond the acute phases of the illness [3]. These impairments in BD (I and II) persist even after significant mood symptoms have remitted. It is estimated that up to 60% of individuals do not recover completely after episodes [4] and only 38% of them achieve functional recovery after a manic phase [5]. This means that work productivity and employment may be negatively influenced [6]. Recovery includes not only symptomatic but also functional or premorbid levels of previous psychosocial functionality, and adaptive social relations. The quality of interpersonal relationships is often mentioned as one of the most important outcomes for patients with BD [7], as social impairment is observed in many patients with this disorder [8, 9]. Almost half of BD I patients and approximately three quarters of those with BD II will first have an episode of depression [10] and they can be misdiagnosed with unipolar depression (UD). This issue may lead to inadequate treatment [11], and this may have clinically relevant consequences. In many cases, it is difficult to distinguish BD from UD, approximately 69% of patients with BD are initially misdiagnosed with UD [12]. BD and UD have similarities and differences; both are chronic and recurrent disorders and both diseases may lead to cognitive and functional impairment [13, 14]. Individuals with UD present depressive episodes only, and those with BD II or I disorder show increasingly pronounced episodes of mood elevation. Another difference refers to the age of onset: patients with BD are younger at onset of first mood episode [15]. Clinical severity is greater among BD patients, as they have a higher prevalence of suicidal ideation [10].

The factors that may play a role in functional recovery of BD have been seldom studied, and understanding facts that might contribute to such inabilities is of essential importance. There is a need to understand mechanisms that may contribute to poor outcome in their psychological functioning. Thus, exploring social cognition in this population is particularly meaningful. Social cognition is an aspect of cognition that relates to the processing of social information for adaptive functioning [16]. Research on social cognition in BD is scarce and investigations have focused mostly on theory of mind (ToM), as deficits in ToM may contribute to deficiencies in social behavior [17, 18]. ToM is the ability to attribute mental states to others’, including beliefs, desires, emotions, and intentions [19], and refers to a competence, that is critical. ToM allows to choose adequate responses for successful interpersonal functioning [20, 21]. ToM enables individuals to decode others’ mental states based on observable information such as facial expressions and bodily gestures [22] and dysfunctions in ToM may be detrimental to social cognitive functioning [23]. There is research related to poor ability in the processing of social information in BD. In comparison to controls, psychosocial impairment is common across the three phases of BD (depression, mania and euthymia) although it has been verified that social functioning in BD patients is poorer in depression and hypomania [24].

For example, Gruber (2011) [25] and Owen et al., (2017) [26] discuss how individuals in manic mood states may be impaired in social interactions due to their increased self-esteem or delusions of grandeur, whereas those in depressive episodes may experience a loss of self-esteem and loss of interest to engage in social interactions. They suggest that symptom severity in either state of bipolar disorder can negatively affect communication in dyadic interactions. Individuals with bipolar disorder have shown impairment in role-playing scenarios of social interactions relative to healthy comparison participants [27, 28]. In attention studies of emotional stimuli, bipolar participants in manic states have a bias towards positive stimuli and positive emotional cues [25, 29], whereas participants in depressed states have a bias for negative emotional cues, e.g., [30, 31].

The literature established that in bipolar disorder some form of ToM impairment has been observed in all mood states, including euthymia [32]. Some studies have shown poor ToM performance in both manic and depressed patients and it varies depending of the state [33]. These authors examined ToM decoding abilities in the three phases of BD and found that they were significantly less accurate in the manic phase. Other studies have shown poor ToM performance in both manic and depressed bipolar patients, but not in patients in remission [34]. Some authors have observed significant ToM dysfunctions in BD remitted patients [35, 36].

Under the specific skills or aptitudes that fall under the general term ToM, this research studied Interpersonal Accuracy (IPA) in remitted BD, as it may have an influence on functional impairment in this population. IPA refers to a perceiver´s ability to decode correctly other person´s states or traits that subsumes specific judgment skills related to emotion, deception, personality or other social characteristics of people [37, 38]. IPA has an adaptive value in workplaces, clinical settings and social life; it contributes to psychological adjustment and adaptive interactions with others [18]. This ability to decode and reason about others’ mental states is critical to successful social and interpersonal functioning and might be related to positive behavioral outcomes [39]. As results of IPA are unclear in remitted BD, we chose this group to try to clarify this issue. When IPA deficits are present, attention should be paid to social adjustment of BD and difficulty in understanding the perspective of others may be an impediment to some psychological interventions.

The majority of research in IPA with BD has focused on facial emotions decoding, and some tests have been employed to measure IPA in this population. For example, the *Reading the Mind in the Eyes test (RMET*) [40], has been widely used in BD with different results. Most authors have found deficiencies in this population, in which stable bipolar participants scored significantly lower than the control group [41, 42, 43, 44]. Other authors have confirmed that there are not statistically significant differences between the performance of euthymic BD and control participants in this test [45, 46]. Another research, also using the RMET, compared remitted BD I and BD II, and both groups obtained similar results than control participants [47].

Social cognition has also been studied in UD, but mostly with facial affect recognition. Some studies have reported deficits in this population, when they are in symptomatic remission, comparing them to control participants [48, 49]. Wolkenstein et al. [50] compared UD to control participants, during an episode. Their results showed that they did not show deficits in facial affect recognition but they had difficulties in integrating contextual information about other people. Nevertheless, there is a lack of behavioral studies in UD in the domains of prosody, body language and IPA to determine if these findings are consistent across all components of social cognition in UD.

Most studies assessing ToM in BD have often lacked a clinical comparison, only a control group was included [23, 51, 52, 53]. To isolate disorder specific versus shared IPA impairments across mood disorders, a comparison with a group of UD was included in this research, using an ecological task, the MiniPONS test [54], a tool that has not been employed either in BD or in UD.

The use of ecologically valid IPA tasks may be an important advance in comparison to the large number of previous studies that have used only static facial stimuli in BD population. Dynamic stimuli approximate to real life situations, as interpretation of nonverbal signals is an important aspect of interpersonal accuracy. Body signals have shown to play an important role in emotion recognition [55], as these sources of nonverbal information are highly relevant in human communication [56]. When using dynamic stimuli, participants can draw upon the whole spectrum of cues as facial expression, body language and voice. Social relationships are favored by the understanding and interpretation of facial, prosodic and emotional cues from the body, and impairments in this area might contribute to the disorder´s psychosocial outcome [53]. The difficulties in nonverbal area may affect negatively quality of life in BD [57]. Vaskinn et al. [58] found a global impairment in the ability of individuals with BD to perceive emotions from bodily movement, strongly associated with functional capacity, and findings of Martino, Samamé and Strejilevich [59] showed that BD, in the recognition of disgust and fear, got worse results than controls. In general, BD shows deficits in social cognition measures with context sensitivity and involvement of real-life scenarios [60]. Tools like the PONS [61] or the MiniPONS (short form of the PONS) [54], have not been utilized either in BD or in UD. Both tests assess the ability to recognize the communication of feelings, attitudes and intentions from nonverbal expressions in face, voice, gestures and body postures and have been employed to verify social effectiveness [62].

### The present study

The current study aimed to examine IPA in patients with BD I and BD II in euthymic state, comparing them with individuals with UD and a group of control participants without psychiatric disorder. The MiniPONS (in its Spanish version) [63] was employed, to examine their ability to understand complex affective states through the perception of different nonverbal channels. We predicted that BD (I and II) would score significantly lower than control participants in the total number of responses. Another objective of this study was to find out if BD groups scored differently than the other groups, depending of the emotional valence of the stimuli. Gruber [25] has shown that BD patients exhibit heightened positive emotion responses; thus, the hypothesis was that persons with BD would give a higher number of correct answers than the healthy controls and UD, in the positive valence items.

## Materials and methods

### Participants

In this study a total of 267 persons participated, 119 had a diagnosis of BD (females = 56, males = 63). Seventy of them were diagnosed with BD I (females = 30, males = 40, mean age = 44.50, SD = 11.50) and 49 with BD II (females = 23, males = 26, mean age = 49.90, SD = 11.50), remitted. For comparison, 39 euthymic individuals diagnosed with UD were recruited (females = 33, males = 6, mean age = 62.90, SD = 9.71) and 119 healthy controls, (females = 65, males = 54, mean age = 46.10, SD = 10.80). UD patients enrolled reported a different number of males and females, there are significantly more women than men. UD sample consisted in voluntary subjects from the outpatients clinic from an hospital in Madrid (Spain). There were more voluntary women than men in this group, but this was accepted, given that there are gender differences in this disorder. The prevalence of depression is twice as high in women as in men [64, 65]. UD age was higher than that of BD groups, as the age of onset of UD disorder is about ten years significantly higher compared to BD [66, 67]. Control participants were the same age and had the same level of education as BD subjects. BD participants were selected as volunteers in two associations of persons affected by bipolar disorder in Madrid.

Demographic and clinical characteristics of the BD and UD samples are summarized in Table 1.

**Table 1:** Demographic and clinical characteristics of BD and UD participants.

	**BD I (n = 70)**		**BD II (n = 49)**		**UD (n = 39)**		**Control (n = 119)**		**F**	***df***	***p***
**Age (Years)**	44.50±11.50		49.90±11.50		62.90±9.71		46.10±10.80		27.80	3; 273	< .001
**Age at onset**	20.23±3.8		26.50±9.3		33.47±8.43		-		46.23	2; 159	< .001
**Gender**	**n**	**%**	**n**	**%**	**n**	**%**	**n**	**%**	**χ*2***	***df***	***p***
Male	40	57.10	26	53.10	6	15.40	65	54.60	12.40	3	.006
Female	30	42.90	23	46.90	33	84.60	54	45.40			
**Medication BD**	**BD I**				**BD II**						
	**n**		**%**		**n**		**%**				
Lithium	25		38.46		12		25.53				
Anticonvulsant	24		36.92		17		36.17				
Antipsychotic	28		43.07		7		14.89				
Antidepressant	10		15.38		12		25.53				

To confirm the diagnoses, the Mini International Neuropsychiatric Interview was administered, in its Spanish adaptation [68]. Inclusion criteria were: having a diagnosis of BD (I or II) or UD and for controls, absence of any lifetime DSM-IV R Axis I diagnosis. The exclusion criteria for BD and UD were as follows: patients with a (hypo)manic or depressive episode in the previous three months, alcohol abuse in the past six months or use of psychoactive substances during the same period. All participants with psychiatric diagnosis (BD and UD) were having pharmacological treatment and had to bring a report from their psychiatrist confirming the diagnosis.

All subjects taking part in this research were Spanish speakers and completed a written consent to participate in the investigation. The study was approved by the Ethics Committee of the Hospital General de la Defensa Gómez-Ulla, in Madrid, and has been carried out in accordance with the Helsinki Declaration of 1975.

### Measures

To assess euthymia, in BD and UD samples, the Beck Depression Inventory (BDI II), in its Spanish version [69], was employed, and Young Mania Rating Scale (YMRS) in its Spanish adaptation [70], in BD sample.

BD and UD patients had to be euthymic as determined by a score ≥ 30 on the BDI II and BDs ≥ 7 on the YMRS as well. The ≥ 30 score was used in BDI II. In the Spanish adaptation for BDI II, for non-clinical and clinical Spanish populations, the cut-off scores would be equal to or higher than 19 and 30 respectively, since those scores would show specificities over 90% and positive predictive values of 61% [71].

### Assessment of IPA

The test MiniPONS [54], in its Spanish version [63] was administered. MiniPONS consists in a set of short video clips that feature a woman with manipulated negative and positive emotional tone of facial expressions, body language, and voice. The MiniPONS is a test of accuracy of inferring the affective meanings of nonverbal cues. In this test, all the stimuli are grouped in a 2x2 design that combines affective valence (positivity-negativity) and dominance (dominance-submission). These categories are represented by three different type of video channels (full figure, neck to knee, and face) and two audio channels. It is administered through a computer application that presents the stimuli and records the responses (the total score). The response procedure consists in asking the subject to select, from 2 possible options, which one he thinks is the correct answer as to what the woman in the films is expressing. In this sample, internal consistency is around .70.

### Statistical analysis

Comparison between groups was done. The software used for the analysis was SPSS version 23 (IBM Corporation, 2015). An alpha significance level equal to 0.05 was used for the analysis. The descriptive statistics for the variables were considered in the present study, to find out if control group had higher scores than clinical groups (BD I, BD II and UD) in the number of right answers, and a better performance in every subscale of the MiniPONS. An ANCOVA test was applied to control the effects of age on the MiniPONS scales. To find out which groups were different, a Tukey`s post hoc test was done, to compare the performance of all clinical groups with the control group.

## Results

Table 2 shows the descriptive statistics for the variables considered in the present study.

**Table 2:** Descriptive statistics.

MiniPONS	Group	N	Mean	Median	SD	Min	Max	Skew	Kurt
Number of right answers	BD I	70	45.10	45.00	4.85	28.00	56.00	-.632	1.62
	BD II	49	45.70	46.00	4.75	33.00	55.00	-.356	-.134
	UD	39	42.70	43.00	4.97	32.00	53.00	-.106	-.585
	Control	119	50.20	50.00	3.70	39.00	58.00	-.338	-.040
Audio prosody channel	BD I	70	10.80	11.00	2.07	3.00	14.00	-1.15	2.77
	BD II	49	11.30	12.00	1.97	6.00	15.00	.400	.097
	UD	39	10.40	11.00	1.97	7.00	14.00	-.051	-.96
	Control	119	12.20	12.00	1.98	6.00	16.00	-.44	.029
Combined channel	BD I	70	12.00	12.00	1.63	8.00	16.00	.012	.350
	BD II	49	11.80	12.00	1.90	7.00	15.00	-.488	.135
	UD	39	11.00	11.00	2.21	6.00	15.00	-.138	-.177
	Control	119	13.20	14.00	1.63	8.00	16.00	-.493	-.157
Face video channel	BD I	70	11.30	11.00	1.68	8.00	15.00	.050	-.82
	BD II	49	11.40	12.00	1.63	8.00	15.00	-.159	.035
	UD	39	11.00	11.00	1.57	8.00	14.00	-.171	-.91
	Control	119	12.50	12.00	1.48	9.00	15.00	-.128	-.46
Body video channel	BD I	70	11.10	11.00	1.81	6.00	15.00	-.525	.204
	BD II	49	11.20	11.00	1.74	6.00	14.00	-.585	.199
	UD	39	10.40	10.00	2.28	5.00	15.00	-.089	-.312
	Control	119	12.30	12.00	1.59	9.00	16.00	-.123	-.247
Positive Valence	BD I	70	22.60	23.00	3.20	14.00	29.00	-.444	.059
	BD II	49	22.90	23.00	2.88	17.00	28.00	-.075	-.740
	UD	39	21.30	22.00	3.28	13.00	28.00	-.364	-.472
	Control	119	25.20	25.00	2.52	19.00	30.00	-.235	.221
Negative Valence	BD I	70	22.60	23.00	2.94	13.00	29.00	-.464	.675
	BD II	49	22.80	23.00	3.11	16.00	28.00	-.374	-.323
	UD	39	21.40	22.00	3.18	10.00	26.00	-1.17	3.00
	Control	119	25.10	25.00	2.45	19.00	30.00	-.309	-.508
Dominant	BD I	70	22.70	23.00	3.11	12.00	29.00	-1.15	2.38
	BD II	49	22.60	23.00	3.23	13.00	29.00	-.491	.763
	UD	39	21.50	21.00	2.95	16.00	29.00	.123	.139
	Control	119	24.90	25.00	2.51	16.00	31.00	-.446	.866
Submissive	BD I	70	22.40	22.50	2.70	15.00	28.00	-.453	.060
	BD II	49	23.10	23.00	2.71	18.00	28.00	.151	-.977
	UD	39	21.20	22.00	3.24	13.00	29.00	-.062	.426
	Control	119	25.40	25.00	2.21	17.00	30.00	-.421	.705

Table 2 summarizes the Number of participants, the Means and the Standard deviations of all the groups.

Results show that control group has higher scores in the number of right answers and a better performance in every subscale of the MiniPONS.

To control the effect of age, an ANCOVA analysis has been performed. Results are shown in Table 3. It is observed that, despite controlling the effect of age on the MiniPONS scales, there are still differences in the performance of each scale associated with the groups (BD, UD and control). The effect size associated with the group is practically double that the effect size associated with age. This happens in in all areas, being the only exception the Audio-prosody channel. In this channel there are differences between BDI and control participants and between UD and controls, but not between BD II and control group. The rest of the differences are not significant.

**Table 3:** ANCOVA summarizes the differences of every group in the scores in different channels.

MiniPONS	Variable	F	df	p	$\eta_{*}^{2}$
Number of right answers	Group	31.50	3, 272	<.001	.258
	Age	25.50	1, 272	<.001	.086
Audio prosody channel	Group	10.33	3, 272	<.001	.102
	Age	6.19	1, 272	.013	.022
Combined channel	Group	13.31	3, 272	<.001	.128
	Age	5.92	1, 272	.016	.021
Face video channel	Group	13.00	3, 272	<.001	.125
	Age	20.50	1, 272	<.001	.070
Body video channel	Group	11.12	3, 272	<.001	.109
	Age	8.67	1, 272	.004	.031
Positive Valence	Group	17.90	3, 272	<.001	.165
	Age	19.00	1, 272	<.001	.065
Negative Valence	Group	18.30	3, 272	<.001	.168
	Age	10.90	1, 272	.001	.038
Dominant	Group	13.80	3, 272	<.001	.132
	Age	12.70	1, 272	<.001	.045
Submissive	Group	28.30	3, 272	<.001	.238
	Age	20.20	1, 272	<.001	.069

Group differences and age in the prediction of MiniPONS scores are in the ANCOVA analysis in Table 3.

Table 4 is the pairwise comparison between groups (with Tukey’s post-hoc test). Results show that, when the clinical groups (BD I, BD II and UD) are compared with the control group, they perform worse, and there are no differences when comparing the clinical groups among them. In the audio prosody channel, there were no differences between patients with BD II and control group.

**Table 4:** Tukey post-hoc comparisons are shown in this table.

MiniPONS	Group	Mean difference	SE	df	t	p
Number of right answers	BD I- BD II	-1.143	.794	272	-1.440	.476
	BD I-Control	-5.279	.635	272	-8.316	<.001
	BD I- UD	.262	.943	272	.278	.992
	BD II- Control	-4.136	.720	272	-5.749	<.001
	BD II- UD	1.405	.952	272	1.475	.454
	Control- UD	5.541	.869	272	6.374	<.001
Audio prosody channel	BD I- BD II	-.675	.374	272	-1.808	.272
	BD I-Control	-1.513	.299	272	-5.064	<.001
	BD I- UD	-.104	.444	272	-.233	.996
	BD II- Control	-.838	.339	272	-2.474	.066
	BD II- UD	.572	.448	272	1.276	.579
	Control- UD	1.409	.409	272	3.445	.004
Combined channel	BD I- BD II	.120	.331	272	.362	.984
	BD I-Control	-1.171	.265	272	-4.422	<.001
	BD I- UD	.608	.394	272	1.544	.412
	BD II- Control	-1.291	.300	272	-4.300	<.001
	BD II- UD	.488	.397	272	1.229	.609
	Control- UD	1.779	.363	272	4.905	<.001
Face video channel	BD I- BD II	-.3557	.287	272	-1.241	.601
	BD I-Control	1.333	.229	272	-5.814	<.001
	BD I- UD	-.443	.341	272	-1.299	.564
	BD II- Control	-.977	.260	272	-3.760	.001
	BD II- UD	-.087	.344	272	-.253	.994
	Control- UD	.89	.314	272	2.835	.025
Body video channel	BD I- BD II	-.231	.331	272	-.699	.897
	BD I-Control	-1.262	.265	272	-4.767	<.001
	BD I- UD	.200	.394	272	.509	.957
	BD II- Control	-1.031	.300	272	-3.434	.004
	BD II- UD	.432	.397	272	1.087	.698
	Control- UD	1.463	.363	272	4.034	<.001
Positive valence	BD I- BD II	-.633	.526	272	-1.203	.625
	BD I-Control	-2.672	.421	272	-6.348	<.001
	BD I- UD	.041	.626	272	.066	1
	BD II- Control	-2.039	.477	272	-4.273	<.001
	BD II- UD	.674	.632	272	1.068	.710
	Control- UD	2.713	.577	272	4.706	<.001
Negative valence	BD I- BD II	-.509	.521	272	-.979	.762
	BD I-Control	-2.607	.416	272	-6.261	<.001
	BD I- UD	.221	.619	272	.357	.984
	BD II- Control	-2.097	.472	272	-4.444	<.001
	BD II- UD	.731	.625	272	1.170	.646
	Control- UD	2.828	.570	272	4.959	<.001
Dominant	BD I- BD II	-.126	.529	272	-.238	.995
	BD I-Control	-2.219	.423	272	-5.247	<.001
	BD I- UD	.214	.629	272	.340	.986
	BD II- Control	-2.093	.479	272	-4.366	<.001
	BD II- UD	.339	.634	272	.535	.950
	Control- UD	2.432	.579	272	4.200	<.001
Submissive	BD I- BD II	-1.017	.472	272	-2.153	.139
	BD I-Control	-3.060	.378	272	-8.099	<.001
	BD I- UD	.049	.562	272	.086	1
	BD II- Control	-2.043	.428	272	-4.771	<.001
	BD II- UD	1.066	.567	272	1.880	.239
	Control- UD	3.109	.517	272	6.008	<.001

## Discussion

The present study examined if IPA deficits in BD were evident in comparison to control group and UD. Four groups were considered in the analysis: BD I, BD II, UD and control group (Tables 2–4). After controlling the effect of age, in the post hoc tests, it is observed that there is an effect of group and age, although the effect of the group is always greater than that of age. With respect to the different channels, in the audio-prosody channel, the control group had the highest score than the other groups, there were significant differences only between BD I and control participants and between UD and controls, but the differences between BD II and control group are not significant. In face video channel, no differences exist between clinical groups, but control group performs better than all the other groups, and these results are repeated in body video channel and in the combined channel. With respect to positive, negative, dominant and submissive channels, there are no differences among clinical groups and control group. This group performed significantly better.

Results showed that, when BDs (I and II) had to evaluate different types of nonverbal cues, with the MiniPONS, their judgements were impaired, as they did not recognize well the implicit understanding of others´ communication. Our hypothesis was that remitted BD would perform worse than controls and UD. Contrary to our hypothesis, results show that BD, in recognition of the meaning of gestures in face, body and voice intonation, performed significantly worse than control group, but not than UD. Researchers have proposed that individuals with depression show reduced positive emotion reactivity [72], unlike BD, that when compared with healthy population, an alteration of positive affect exists in this group of patients [73].

Affective intensity can be an indicator of impairment beyond the predictive value of inter-episode symptoms in this population [74]. Our hypothesis was that BD groups would give a higher number of correct responses in positive valence items. However, this result did not come about. In this study, BD individuals presented significant lower overall results than control group in the evaluated IPA task, including recognition of positive valence stimuli, which can be understood as an empathy deficit. This emotional deficiency might be one of the mechanisms that can facilitate the maintenance of maladaptive behaviors in this disorder. Social cognitive deficits and functional impairment are associated with poor psychological adjustment in BD [57, 75]. Body and facial expressions and body postures display relevant information about emotional behaviors and intentions [76], and research exists that shows that deficits in facial emotional identification have a negative impact on participation to social and daily activities and hobbies in BD [77]. For these group of patients, the ability to accurately infer the emotions of others, to identify and anticipate the reactions and intentions of other individuals is highly important in social situations, as it provides information on the meaning of their responses to forthcoming events. Treatment modalities of BD comprise psychotropic medication and psychosocial interventions [78]. As Gitlin and Miklowitz [2] suggest, in BD, recovery comprises symptomatic and functional improvement and psychosocial interventions can speed recoveries from episodes [79]. A desirable goal to accomplish should provide treatment to achieve syndromal as well as functional recovery in BD. BD treatment, then, presents challenges in preventing recurrences and assuring complete recovery between episodes in terms of both symptom remission and restoration of social and occupational functioning. Prevention and treatment programs are needed in the development of future interventions in BD, involving comprehension of the correct complex affective states. According to the model of emotional competence [80], people with higher scores in the test MiniPONS may have more social sensibility skills, as they can easier perceive internal states of others. Recognition of dynamic nonverbal signals as face and body movements are relevant in human communication [56] and a high level of proficiency in the recognition of nonverbal signals relates to the ability to correctly infer the complex affective states that individuals experience and communicate in specific social situations.

Acquirement of competences in the inference of others´ internal states should help BDs to a better understanding of social communication and thus, increase the satisfaction in interpersonal relationships. To accomplish the objective of functional rehabilitation of BD patients, achievement of such abilities might have a greater impact in their social functionality and, consequently, lead to a higher adaptation to everyday life.

There are some limitations in this research and some issues of this study remain unresolved. The “MiniPONS” is merely one of the methods that can be used to determine deficits in IPA. In this research, the MiniPONS was chosen because it has ecological validity, as it presents dynamic scenes that are closer to real life situations than static pictures. Although there are numerous advantages in using this test, the fact that videos in this include only features of a woman make results to be generalizable only for woman features, and more research is needed in the case of men features. Among the main limitations and directions for future research, there is the fact that this research has been done with remitted BDs, and it would be interesting to incorporate patients in other phases of the disease, to examine the effects of mood. Comparison between BD-mania, BD-depression and BD-euthymia is important and can be a future prospect. Another limitation is that, in this research, all BD participants were medicated, but we were unable to investigate the influence of medication effects on results. As to whether these drugs worsen social cognitions, such as IPA, further research is required. The medication profile of this sample was markedly heterogeneous. Dose effects, or the type of medication might have potential influences of medication on IPA performance. A future prospective is to control this variable in further studies. Besides, comparing them with other groups that present IPA deficits such as schizophrenia and autism, could be a line of future research. Another future prospect is to conduct a longitudinal study with chronic BDs and analyze if there are changes in their interpersonal accuracy.

### Conclusions

This research studied ToM in BD, with an IPA measure based on social situations of different types of activity, the test MiniPONS. Results showed impairments in the performance of BD and UD, in the recognition of the meaning of gestures in face, body and voice intonation. Acquirement of competences in the inference of others´ internal states should help BDs to a better understanding of social communication. Thus, treatment modalities involving a better comprehension of others’ internal states, is a desirable goal to achieve in this population.

## Supporting information

S1 Table Raw data of patients participating in this study. Clinical characteristics of patients and results in the different scales of MiniPONS.
(CSV)
